# Multivariate Genome-Wide Analysis Delineates Shared Genetic Determinants Across Periodontal and Oral Microbial Phenotypes

**DOI:** 10.3290/j.ohpd.c_2823

**Published:** 2026-10-21

**Authors:** Yamei Zhang, Mengshan Chen, Fang Zhang

**Affiliations:** a Yamei Zhang Researcher, Department of Orthodontics, Changsha Stomatological Hospital, Changsha, Hunan, China. Conceived and designed the study, performed analyses, and drafted the manuscript; read and approved the final manuscript.; b Mengshan Chen Researcher, Department of Orthodontics, Changsha Stomatological Hospital, Changsha, Hunan, China. Conceived and designed the study, performed analyses, and drafted the manuscript; read and approved the final manuscript.; c Fang Zhang Researcher, Department of Orthodontics, Changsha Stomatological Hospital, Changsha, Hunan, China. Supervised the study, provided critical revisions, and handled correspondence; read and approved the final manuscript.

**Keywords:** genomic SEM, host–microbiome axis, multivariate GWAS, periodontitis microenvironment, pleiotropy

## Abstract

**Background:**

Periodontitis affects approximately 10% of the global population, posing a serious threat to tooth retention and quality of life. The disease emerges from bidirectional host–microbiome interactions that can shift from homeostasis to dysbiosis and chronic inflammation, yet conventional single-phenotype analyses cannot resolve pleiotropic loci acting across interrelated periodontal and microbial traits. We aimed to jointly model the shared genetic structure of six periodontitis-related phenotypes and to prioritise pleiotropic loci with translational relevance.

**Methods and Materials:**

We harmonised European-ancestry genome-wide association studies (GWAS) summary statistics for six traits representing host periodontal phenotypes (gingivitis, chronic periodontitis, periapical abscess, dental calculus) and oral microbial composition (genus *Porphyromonas*, order *Bacteroidales*). A common genetic factor was estimated using genomic structural equation modelling (genomic SEM), and factor-level genome-wide association analysis was conducted on 4,899,781 SNPs. The resulting signals were annotated through locus discovery and novelty assessment (FUMA), fine-mapping (SuSiE and FINEMAP), gene-level testing (MAGMA), and transcriptome-wide association with Bayesian gene prioritisation (FUSION and FOCUS). Heritability was partitioned across functional annotations (stratified LD score regression), and cell types (CELLECT); spatial enrichment was assessed using gsMap, and a polygenic risk score was constructed with PRS-CS.

**Results:**

A single-factor model provided adequate fit (CFI = 1.0). Factor-level GWAS identified 127 genome-wide significant SNPs consolidating into 56 independent lead variants, all uniquely identified at the factor level. MAGMA prioritised *RASAL2* and *C5orf27*. Fine-mapping yielded 56 variants with posterior inclusion probabilities exceeding 0.95. Transcriptome-wide association and FOCUS converged on the long non-coding RNA *RP11-563N4.1* (PIP = 1). Cell-type enrichment highlighted a vascular–stromal axis led by hepatic sinusoidal endothelial cells, with additional signals in pericytes, cardiac and smooth muscle cells, endocardium, and lung stroma. Spatial mapping localised the strongest aggregate signal to lung tissue, consistent with an oral–lung mucosal axis. Polygenic risk was distributed unevenly across chromosomes.

**Conclusions:**

These analyses delineate a vascular–matrix-centred genetic architecture shared across periodontal and oral microbial traits. The prioritised loci and effector genes provide genetically informed candidates for risk stratification and for targeted investigation of periodontal pathobiology. Clinically, these genetically informed candidates may aid in identifying individuals at elevated risk and in guiding mechanism-based preventive and therapeutic strategies for periodontitis.

The periodontitis microenvironment (PME) is defined as a complex ecosystem sculpted by the dynamic interplay between multispecies oral biofilms and the host immune-inflammatory response. Its collective function, governed by a positive feedback loop that operates across microbial and host determinants, dictates the maintenance of oral tissue homeostasis versus dysbiosis and ultimately drives disease initiation and persistence.^19^ For instance, host–microbial nitrate metabolism modulates nitric oxide–dependent microbial killing and immunoregulation at the periodontal interface, helping to steer the community between eubiosis and dysbiosis.^34^ However, systems-level characterisation of the key causal chains within this microenvironment faces substantial challenges arising from the complexity of the oral–systemic network. Accumulating evidence indicates that these connections are not only bidirectional but also multimodal, interweaving multiple comorbidities and intricate signalling pathways.^16^ Given the considerable global burden of periodontitis–affecting approximately 10% of the world’s population and more than 40% of adults in the United States–and its serious threat to tooth retention and patient quality of life,^18^ with complete tooth loss further associated with elevated all-cause mortality,^7^ clarifying its integrative structure at the systems level is of urgent clinical and public health importance to deepen disease understanding, enable precise identification of high-risk individuals – an objective for which self-report-based screening algorithms have recently been developed and validated^8^ – and optimise prevention and treatment strategies.

Contemporary understanding is grounded in an ecological framework of polymicrobial synergy and dysbiosis, wherein keystone pathogens together with sustained gingival inflammation drive a proteolysis–inflammation–pathogen enrichment positive feedback loop.^27^ Yet the prevailing research paradigm has fundamental limitations. First, although traditional studies frequently partition analyses by clinical status, microecological evidence supports a disease continuum linking gingivitis and periodontitis. At the same time, direct, cross-state comparisons spanning health–gingivitis–periodontitis remain limited, which helps explain methodologically why the evolution of the PME is difficult to fully characterise within a single-phenotype framework.^1^ Likewise, endodontic–periodontal lesions and dental calculus are often treated as separate entities or local triggers in existing classifications or consensus statements, and specific bacterial genera are commonly discussed in isolation in microbiome reviews rather than being integrated into a unified microenvironmental model.^14^ Second, core causal relationships remain unresolved. Foundational questions – whether dysbiosis is a cause or consequence of disease, and which determinants govern progression from gingivitis to periodontitis – still rely predominantly on cross-sectional associations, lacking definitive causal inference.^4^ This fragmentation of evidence, coupled with causal uncertainty, underscores the need for a systems-analytic framework capable of integrating multiple phenotypes and resolving their shared structure.

Recent syntheses of genetic risk variants have implicated impaired maintenance and repair of periodontal tissues as causal mechanisms in periodontitis,^35^ underscoring the value of genetic approaches for dissecting disease aetiology. Mendelian randomisation has provided proof-of-concept causal evidence in specific domains, yet its single-exposure–single-outcome design yields unstable or conflicting results when extended across the broader oral–systemic network.^20,23,24,51
^ Functional genomic approaches such as TWAS, colocalization, and fine-mapping link loci to genes but remain organised around single phenotypes,^41^ while single-cell multi-omics and animal models illuminate cellular mechanisms without recapitulating the full complexity of human disease at the population scale.^13^ The common limitation is that these methods analyse traits individually and were not designed to quantify the shared genetic architecture across multiple, interrelated phenotypes. Consequently, there is a need for an analytic framework that, within a unified model, jointly characterises the shared genetic structure and latent common factors of the PME.

Accordingly, the aim of this study was to jointly model the shared genetic architecture of six periodontitis-related phenotypes within a single multivariate framework, and to prioritise pleiotropic loci and candidate effector genes with potential translational relevance. To achieve this, we introduce genomic structural equation modelling (genomic SEM), a multivariate framework that integrates genome-wide association studies (GWAS) summary statistics across multiple traits to model and quantify shared genetic structure at the genome-wide level.^12^ Our work makes two principal contributions. First, we construct a genetic network model of the PME that integrates six core elements – gingivitis, chronic periodontitis, periapical abscess, dental calculus, *Bacteroidales*, and *Porphyromonas* – thereby systematically delineating shared and trait-specific genetic architectures within a unified framework. Second, leveraging the latent genetic factors identified by this integrative model, we conduct multivariate GWAS to discover key loci exerting pleiotropic effects on the microenvironmental network and, through downstream functional annotation and pathway enrichment, elucidate the underlying biological mechanisms, providing new genetic evidence and candidate targets to inform future risk stratification and targeted interventions.

## Methods and Materials

### GWAS Summary Statistics Data Sources

To construct our model, we curated publicly available GWAS summary statistics for six traits of European ancestry. These traits were selected to comprehensively represent the PME. They included six related phenotypes: gingivitis, chronic periodontitis, periapical abscess, dental calculus, genus *Porphyromonas* and order *Bacteroidales*. All contributing studies had received appropriate ethical approval and participant informed consent. Complete details for each GWAS, including specific sources and sample sizes, are provided in Table S1.

### Quality Control of Input GWAS Data

A stringent, standardised quality control (QC) protocol was applied to all six sets of summary statistics. This ensured data integrity and compatibility for joint analysis. Using the 1000 Genomes Project Phase 3 European reference panel as a common variant backbone, we performed several steps for each data set. These steps included removal of nonbiallelic and nonautosomal SNPs, filtering out variants with a minor allele frequency (MAF) less than 0.01, exclusion of indels and variants with ambiguous alleles such as AT or CG pairs or mismatched alleles compared to the reference panel, and removal of all variants within the extended major histocompatibility complex (MHC) region on chromosome 6 from 25 to 35 Mb.

### Genomic SEM Construction

We implemented the genomic SEM methodology using the GenomicSEM R package version 0.0.5. This allowed us to model the genetic architecture of the PME. The analysis proceeded in two main stages.^12^ In stage 1, we first ran multivariate LD Score Regression (LDSC) on the six QC-harmonised GWAS summary statistics. This step generated a six-by-six genetic covariance matrix denoted S and its corresponding sampling covariance matrix denoted V. The diagonal elements of S represent the SNP-based heritability (h2SNP) for each trait. The off-diagonal elements represent the genetic covariance between pairs of traits. These results are in Table S2 (supplementary material is available directly from the author). In stage 2, we specified a common factor model. A single latent variable termed the PME was hypothesised to underlie the shared genetic variance across all six indicator traits. This model was fitted to the covariance matrices generated in stage 1. For the factor-level GWAS, each SNP was added to the structural model as an exogenous predictor of the common factor. The genetic covariance matrix (S) and its sampling covariance matrix (V) were expanded to include the covariances between the SNP and each indicator trait, and the SNP effect on the latent factor, together with its standard error, was estimated by weighted least squares. A χ^2^ test of this effect yielded the factor-level association statistic for each variant. Model fit was rigorously evaluated using multiple standard indices. These were the chi-square test (χ^2^), Akaike information criterion (AIC), comparative fit index (CFI), and standardised root mean square residual (SRMR). The results are in Tables S3 and S4. This model allowed us to proceed to a genome-wide analysis of the latent factor. We then calculated the association of each SNP with this common factor. We removed any SNP with a Cochran’s Q statistic *p* < 0.05 from the final factor GWAS results.

### Identification and Novelty Assessment of Genomic Loci

We utilised the FUMA (Functional Mapping and Annotation of GWAS) platform version 1.5.0. This was to identify independently significant genomic risk loci from the latent factor GWAS results. SNPs passing the genome-wide significance threshold *p* < 5 × 10^–8^ were clumped based on linkage disequilibrium r^2^ < 0.1 within a 250 kb window. The most significant SNP in each region was designated as the lead SNP. We employed a GWAS minus locus strategy to determine the novelty of these loci. Each identified locus was cross-referenced against the significant loci from the six original input GWAS summary statistics. A locus from our latent factor GWAS was considered novel if its lead SNP was located more than 1 Mb away from any genome-wide significant SNP in the input GWASs. Further, all lead SNPs were queried against the GWAS Catalog. Gene-based and gene-set enrichment analyses were performed using MAGMA.

### Fine-Mapping to Pinpoint Causal Variants

We performed statistical fine-mapping to dissect the identified risk loci. For each locus, we applied two complementary Bayesian methods, SuSiE and FINEMAP. These were implemented within the echolocatoR R package version 2.0.3. We used a 500 kb window centred on each lead SNP and the 1000 Genomes European LD reference panel. These methods calculate a posterior probability (PP) of causality for each variant. Variants with mean PP > 0.95 within 95% credible sets were prioritised as the most likely causal SNP.

### TWAS for Causal Gene Identification

We conducted a cross-tissue TWAS based on sparse canonical correlation analysis (sCCA-TWAS) to link genetic variants to gene function. The study identified genes whose genetically predicted expression levels are associated with the PME. We employed the FUSION software suite. We leveraged precomputed eQTL weights from 49 tissues in the Genotype Tissue Expression (GTEx) project version 8. An sCCA-based model was used to build expression prediction models. To distinguish true causal genes, we applied FOCUS to the significant TWAS loci. FOCUS computes a posterior inclusion probability (PIP) for each gene in a locus. Genes with a PIP > 0.8 were prioritised as the most likely causal functional effector genes.

### Cell-Type Annotation and Heritability Partitioning

To identify specific cell types where the genetic heritability of PME is concentrated, we employed CELLECT. This method integrates GWAS summary statistics with scRNA-seq data. We utilised the comprehensive Tabula Muris mouse atlas, which profiles ~100,000 cells across 20 tissues.^36^ CELLECT applies S-LDSC to test whether heritability is significantly enriched in genes specifically expressed in particular cell types.^42^ Furthermore, we used S-LDSC with the baseline-LD model (v2.2) to partition PME heritability across 53 broader functional genomic annotations, identifying the contribution of elements such as promoters, enhancers, and conserved regions to the trait’s genetic variance.

### Spatially Resolved Mapping of Trait-Associated Cells

We employed the gsMap pipeline to understand the tissue architecture and spatial context of the implicated cell types. This method integrates GWAS summary statistics with spatial transcriptomics data to map the spatial distribution of trait-associated cells. We leveraged a publicly available spatial transcriptomics data set of a relevant developmental mouse model. By correlating the genetic signal for the PME with the spatial gene expression profiles, gsMap allowed us to visualise the anatomical localisation of cells that are likely critical to the disease process. A mouse–human gene homolog mapping file was utilised for cross-species analyses.

### Polygenic Risk Score Construction

Finally, we constructed a PRS to create a tool for quantifying an individual’s genetic predisposition to the PME. We used the PRS-CS algorithm. This is a Bayesian regression framework that infers posterior SNP effect sizes by placing a continuous shrinkage prior on them. It is informed by LD information from the 1000 Genomes European reference panel. These posterior effect sizes were then used to assess the contribution of each chromosome to the overall genetic risk of the PME.

## Results

### Structural Equation Model Fitting

The six traits were included as indicators of the latent factor based on their genetic covariance structure (Table S2). Genetic covariances between trait pairs are presented in Table S3 and Figure 1. A single common factor model was applied to the empirical covariance matrix, yielding a model fit of CFI = 1 (Table S4). Standardised factor loadings and residual variance estimates are provided in Table S5. The final genomic SEM yielded a GWAS based on 4,899,781 SNPs, effectively representing the PME-related genetic construct.

**Fig 1 Fig1:**
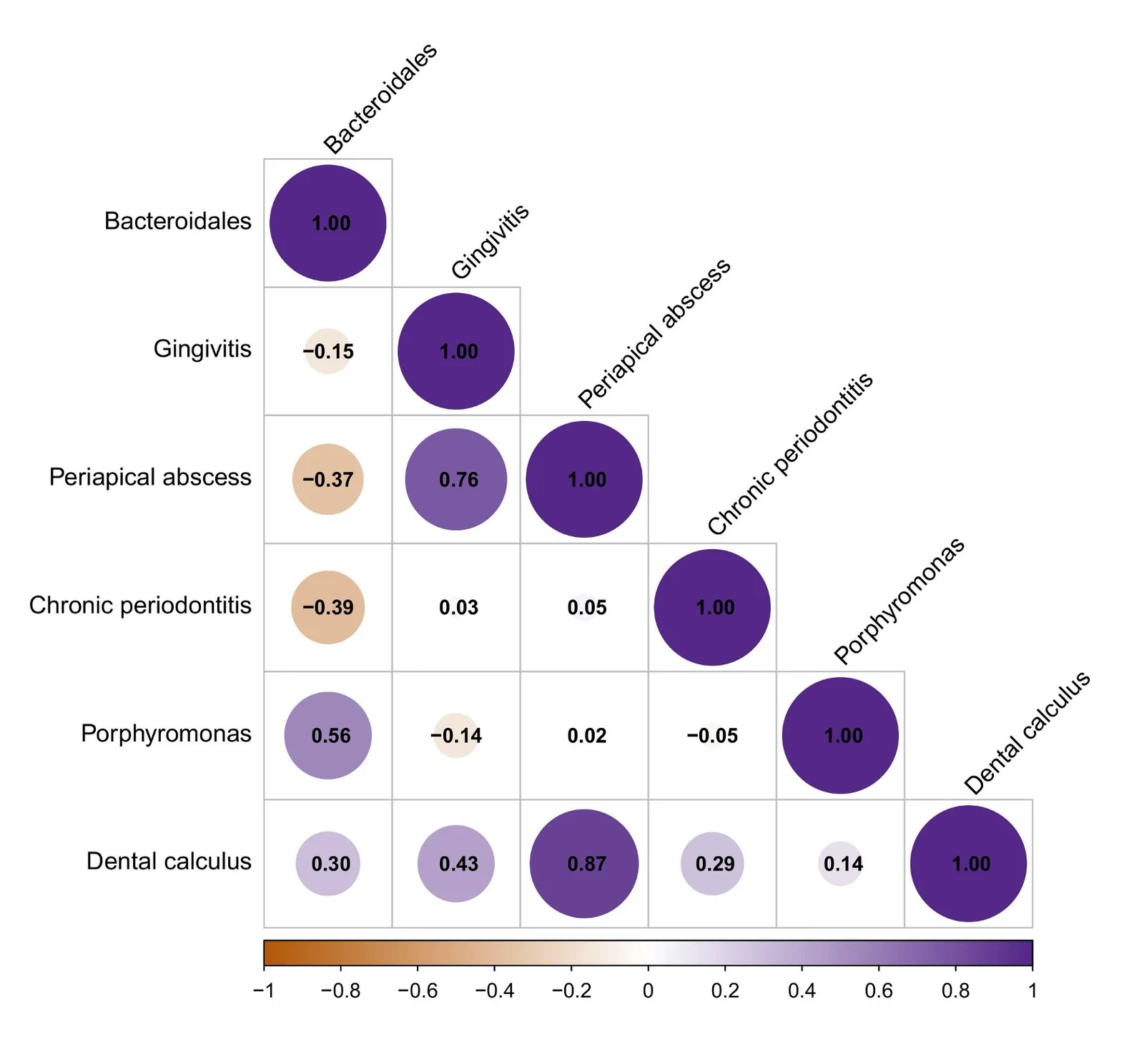
Genetic correlation matrix of PME. The colour intensity and circle size represent correlation strength, ranging from − 1 to + 1.

### Risk Genetic Loci

The genomic SEM-based GWAS for the common factor identified 127 significant SNPs (*p* < 5 × 10^–8^) (Table S6, Fig 2). Further analysis delineated 56 lead SNPs (r^2^ < 0.1) (Table S7). FUMA annotation revealed that these loci are mainly located in intergenic (48.5%) or intronic (36.5%) regions. Novelty analysis identified 56 novel SNPs that had not reached genome-wide significance in the input GWASs (Table S8). GWAS Catalog queries revealed pleiotropic loci (Table S9). For example, lead SNP rs11603665 is associated with hip circumference adjusted for BMI, rs13163351 is associated with macular thickness, and rs73201772 is linked to mean corpuscular volume and mean reticulocyte volume. MAGMA analysis identified two significantly associated genes, *RASAL2* (Z = 6.142, FDR *p* = 7.43 × 10-06) and *C5orf27* (Z = 4.461, FDR *p* = 0.037) (Table S10, Fig 3).

**Fig 2 Fig2:**
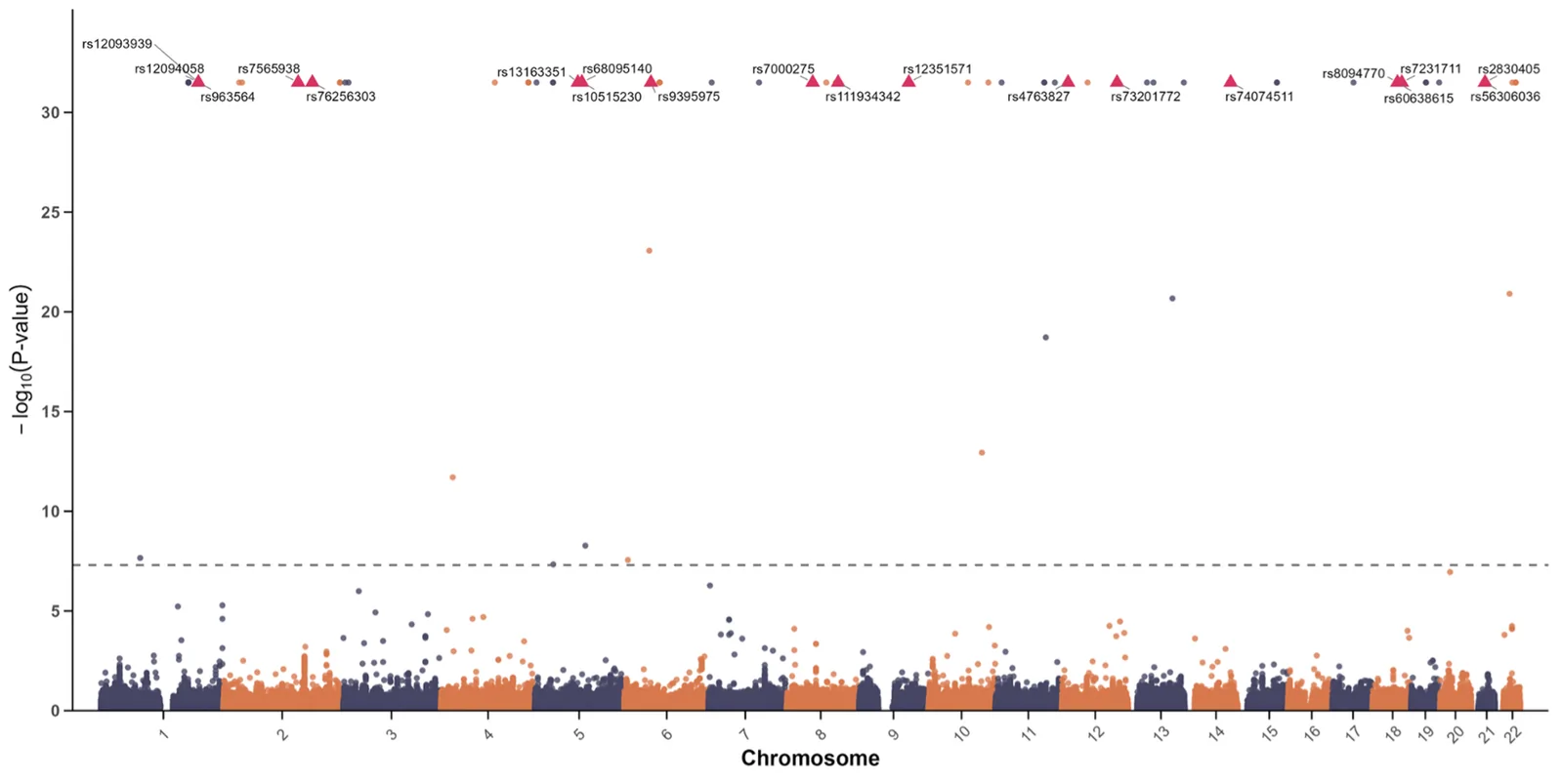
Manhattan plot of novel Genomic-SEM results for PME. The x-axis denotes chromosomal positions, while the y-axis represents the negative logarithm of the *p* value (− log10(P)). The dashed line indicates the genome-wide significance threshold at *p* = 5 × 10−8.

**Fig 3 Fig3:**
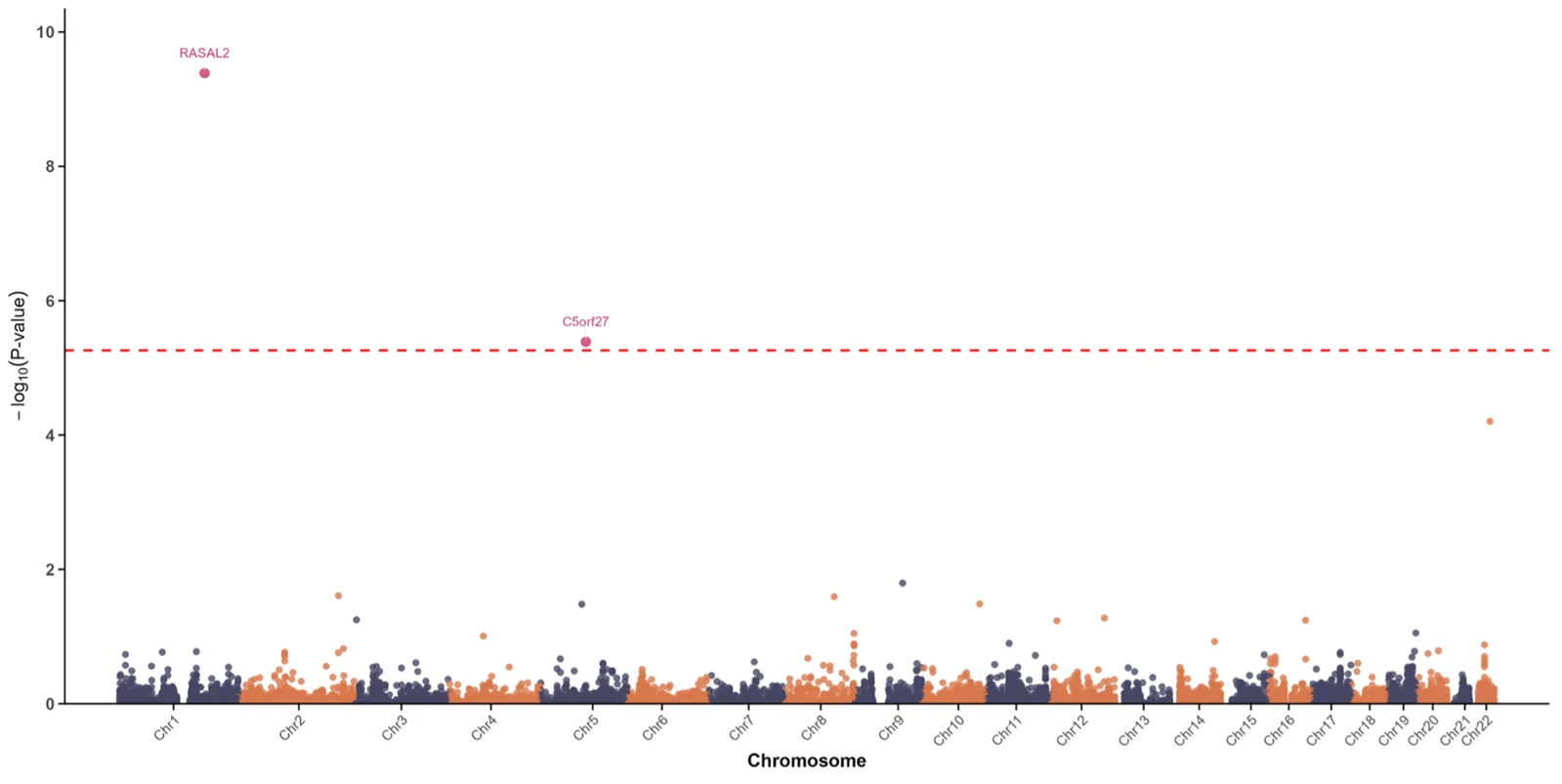
Manhattan plot of GWAS results for PME from MAGMA analysis.

### Fine-Mapping

Fine-mapping identified 56 potential causal SNPs with high PP (PP > 0.95). Representative signals include rs963564 (located in locus NEK7) (GWAS *p* = 2.06 × 10^–^66, T-statistic = 17.215), rs2917929 (locus RGS10) (GWAS *p* = 1.63 × 10^–^46, T-statistic = –14.320), and rs111934342 (locus ATP6V1C1) (GWAS *p* = 5.79 × 10^–^69, T-statistic = –17.552), suggesting these lead SNPs are likely causal variants (Table S11, Fig 4).

**Fig 4 Fig4:**
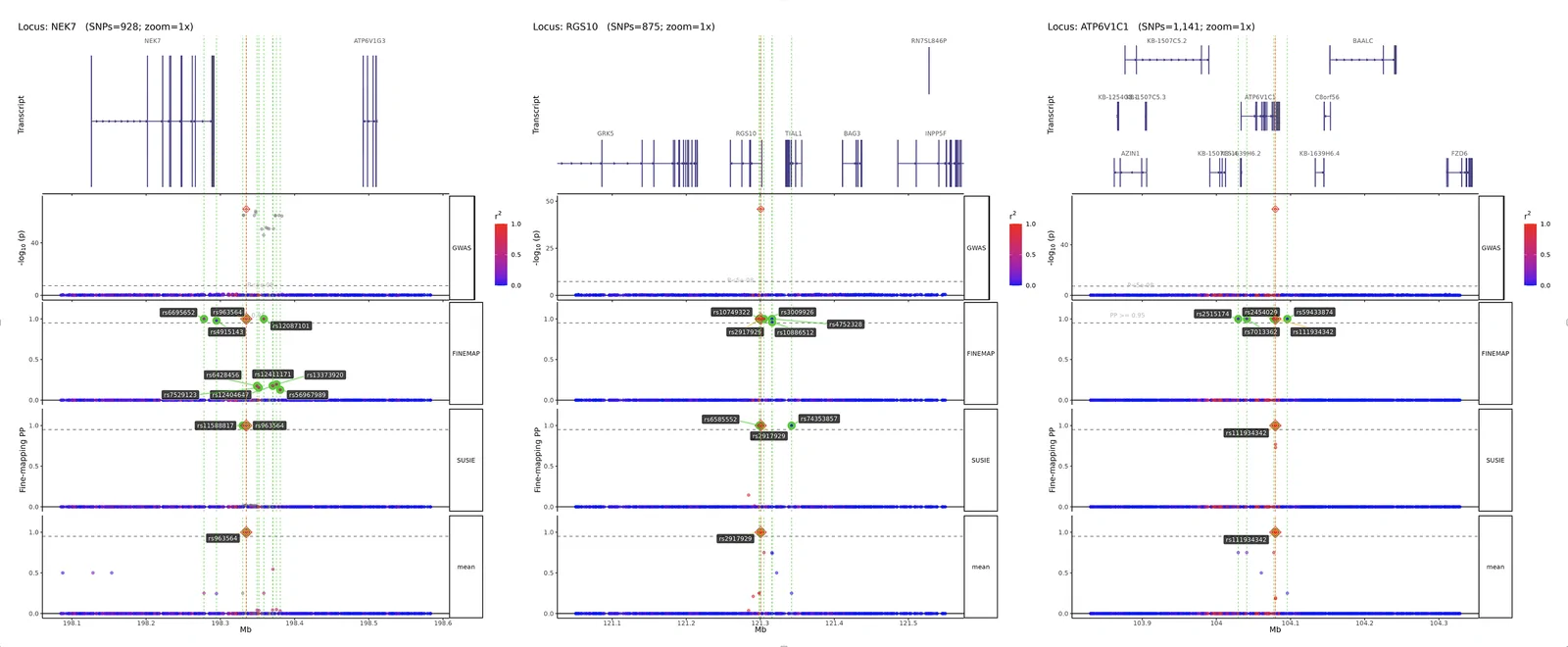
Fine-mapping results of genomic loci with strong associations (PP > 0.95) identified by FINEMAP.

### Gene-Level Identification of Susceptibility

SCCA-TWAS identified one gene surpassing the FDR threshold. FOCUS analysis also identified one causal gene with a PIP > 0.8 (Fig 5). Cross-referencing TWAS and FOCUS results yielded one high-confidence gene-level association (Table 1). The identified gene, *RP11-563N4.1*, was supported by both analyses (Z = 7.35, TWAS *p* = 1.92 × 10^–13^, FOCUS PIP = 1).

**Fig 5 Fig5:**
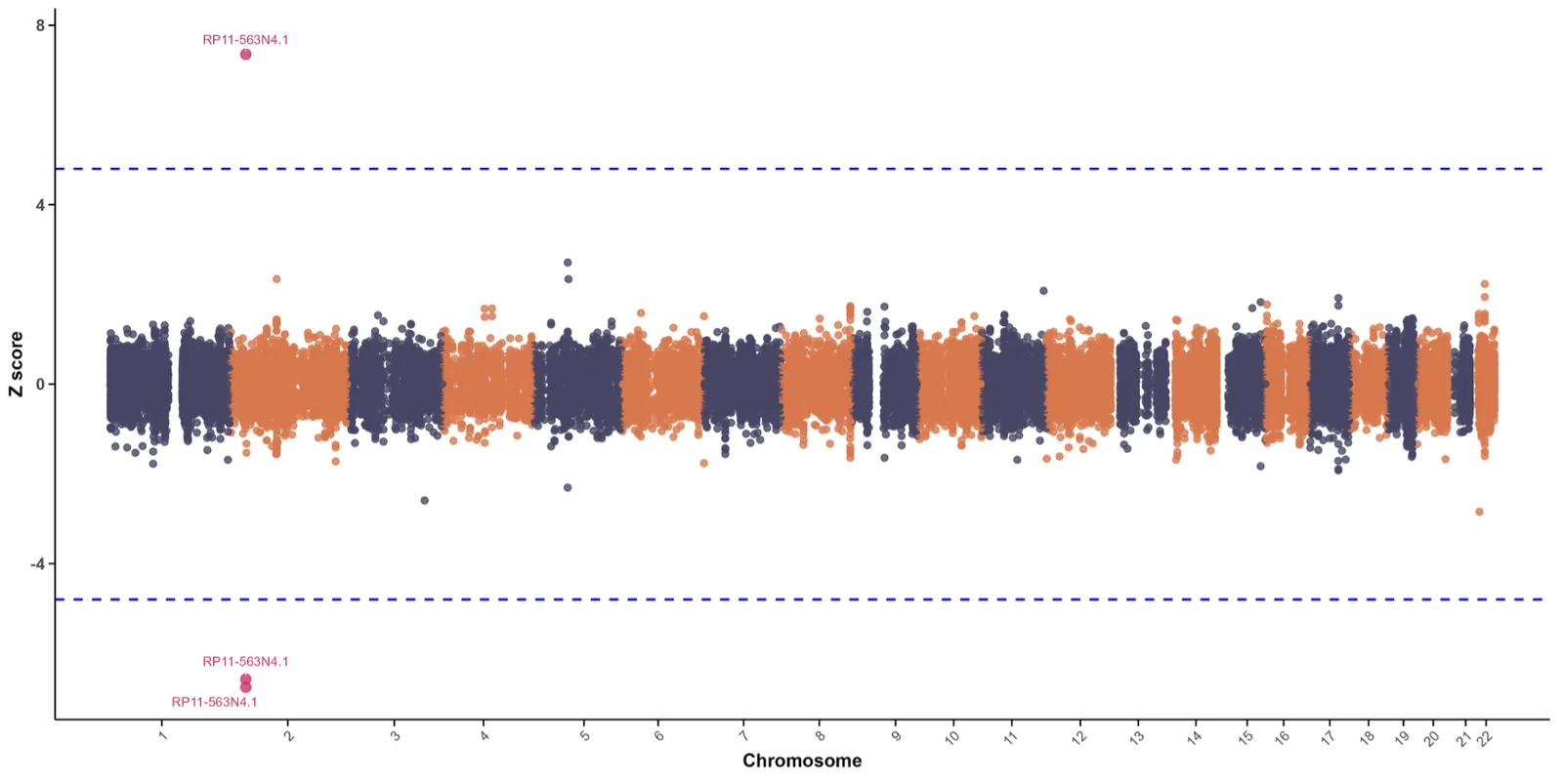
Manhattan plot of results from sCCA-TWAS analysis for PME. The x-axis represents chromosomes, and the y-axis displays the Z-scores.

**Table 1 Table1:** Genetic associations with PME in sCCA and FOCUS analysis

Gene	CHR	Heritability squared	TWAS Z	TWAS FDR	FOCUS pip
*RP11-563N4.1*	2	0.268	7.354307	1.92E-13	1
*RP11-563N4.1*	2	0.1715	–6.57671	4.81E-11	1
*RP11-563N4.1*	2	0.1923	–6.7559	1.42E-11	1


### Cell-Type Enrichment Analysis and Spatially Mapping

Cell-type enrichment analysis identified several cell types showing significant associations with the PME-related genetic construct (Table S12). The strongest association was observed in liver endothelial cells of the hepatic sinusoid (*p* = 0.012). Other significantly associated cell types included brain pericytes (*p* = 0.021), heart cardiac muscle cells (*p* = 0.021), heart smooth muscle cells (*p* = 0.022), heart endocardial cells (*p* = 0.026), and lung stromal cells (*p* = 0.031). Spatial mapping analysis integrating GWAS results with tissue expression data was performed to localise the genetic signal (Table S13). The results showed the strongest association in the lung (*p* = 0.014).

### Heritability Partitioning and PRS

To further characterise the genetic architecture, we partitioned the heritability using S-LDSC across 53 predefined genomic functional categories (Table S14). This analysis did not reveal any functional annotations with statistically significant heritability enrichment after multiple testing correction. Subsequently, a PRS was constructed from the summary statistics to aggregate the cumulative genetic risk. An analysis of its chromosomal contributions showed that chromosome 1 had the highest total PRS sum (15.54), followed by chromosome 2 (14.64) and chromosome 3 (12.37), indicating that the polygenic risk is distributed unevenly across the genome (Table S15).

## Discussion

In this study, we applied genomic SEM to six periodontal and oral microbial phenotypes to model their shared genetic architecture. A single common factor captured the heritable signal shared across traits, and factor-level GWAS identified 56 independent lead variants that were not detected in the contributing univariate GWASs. Downstream fine-mapping, gene-level testing (MAGMA), and transcriptome-wide prioritisation (TWAS and FOCUS) converged on a set of candidate effectors, while cell-type and spatial enrichment analyses localised the aggregate signal to vascular and mural lineages and to lung tissue. These analyses were aimed at characterising the genetic determinants shared across periodontal and microbial traits rather than at establishing causal mechanisms. The central contribution of this work is a genetically informed map of pleiotropic loci and candidate genes acting across the periodontitis-related phenotype network, complementing prior single-phenotype studies that could not resolve genetic covariance across traits. Together, these findings provide genetically prioritised candidates for risk stratification and for the targeted investigation of periodontal pathobiology.

Our fine-mapping analyses implicate key biological pathways closely linked to dysbiosis within the PME. These pathways centre on inflammasome activation, osteoclast-mediated bone resorption, and coupled dysregulation of immunometabolic and mechanotransductive signalling–findings that align closely with established pathobiological evidence in periodontitis. It should be noted that the genes discussed below were nominated through positional annotation of the genes harbouring or nearest to the fine-mapped credible-set variants, rather than through gene-level statistical testing. They therefore represent a lower tier of evidence than *RASAL2* and *C5orf27*, which reached gene-level significance in MAGMA, and *RP11-563N4.1*, which was supported by both transcriptome-wide association and Bayesian fine-mapping. The mechanistic interpretations that follow should accordingly be regarded as hypothesis-generating. At the level of inflammatory regulation, a high-confidence signal at the NEK7 locus provides a mechanistic anchor consistent with periodontal pathology. NEK7 functions as a licensing and bridging factor for the NLRP3 inflammasome, structurally linking adjacent NLRP3 subunits to assemble the NLRP3–NEK7–ASC activation complex. Aberrant inflammasome activation has been shown to drive alveolar bone loss and to shape a microenvironment characterised by heightened osteoclast activity, diminished osteogenesis, and elevated inflammatory tone.^11,25,38
^ Concordantly, the significant association at the *RGS10* locus points to critical regulation of inflammatory osteolysis. External evidence suggests lesion-dependent functions of *RGS10*. In apical periodontitis, *RGS10* inhibition exacerbates alveolar bone destruction and augments NF-κB activation, whereas in bacteria-induced periodontitis, downregulation of *Rgs10* concomitantly reduces inflammatory infiltration and osteoclast activity, thereby protecting bone.^47,49
^ With respect to bone homeostasis, genetic signals converge on osteoclast function and mechanotransduction. The association with *ATP6V1C1* is particularly salient. *ATP6V1C1* encodes the V1-C1 subunit of the vacuolar H+-ATPase (V-ATPase), localises to the F-actin ring in osteoclasts, and is essential for proton pump assembly and acidification. Loss of function attenuates acidification of the resorption lacuna and reduces resorptive efficiency. Conversely, excessive osteoclastic bone resorption is a defining feature of jawbone pathology in periodontitis.^33^ The V-ATPase/*ATP6V1C1* axis is not only mechanistically well defined but also therapeutically tractable. In murine models of periodontitis and apical infection, AAV-shRNA targeting *ATP6V1C1* markedly reduced bone loss by approximately 70%, respectively, while concurrently suppressing local inflammation. Sustained operation of this acidification machinery further depends on Rab32/38-mediated biogenesis of secretory lysosomes and proper targeting of the V-ATPase a3 subunit.^22,32,45
^ On the mechanotransduction front, strong signals at *TEAD1* and *STK3* are concordant with abundant evidence defining the Hippo–YAP or TAZ–TEAD hub as a core module that translates mechanical cues into biochemical outputs, thereby shaping a proliferation–inflammation–bone remodelling niche. YAP/TAZ sit at the centre of this mechanotransductive network, integrating signals from the dental pulp and periodontal tissues. *YAP1* has been identified as a master regulator of periodontal mechanobiology and participates in periodontitis-induced bone loss. Cytoskeleton-to-nucleus coupling via the LINC complex promotes YAP nuclear translocation, effectively driving proliferation and periodontal tissue remodelling.^5,9,50
^ Regarding immunometabolic coupling, our findings highlight a genetic basis for metabolic pathway perturbation. In lipid–inflammation coupling, *SREBF2*, the master transcription factor for cholesterol biosynthesis, can be chronically activated under disrupted cholesterol trafficking. Periodontal pathogens such as *Fusobacterium nucleatum* simultaneously enhance lipid uptake or deposition and inflammatory cascades in macrophages, collectively implicating a cholesterol homeostasis–inflammatory microenvironment coupling as a key route through which *SREBF2* variation may modify periodontal risk.^6,29,39
^ Along the mitochondria–immune axis, the strong association with PPARGC1A underscores the importance of mitochondrial function. In oral lesions, downregulation of PGC-1α coincides with mitochondrial dysfunction and elevated mtROS. Activation of the SIRT1–PGC-1α deacetylation–metabolic axis enhances oxidative metabolism and suppresses NF-κB–mediated inflammation. These observations suggest that PPARGC1A variants may influence alveolar bone destruction by modulating a mitochondria–ROS–inflammation coupling within the periodontal microecosystem.^3,17,44
^


To translate GWAS associations into function and prioritise effector genes, we implemented an integrated post-GWAS pipeline that combined MAGMA, TWAS, and FOCUS. This approach converged on two complementary pathogenic axes, *RASAL2* to Ras/MAPK and *C5orf27/SMIM3* to PI3K–AKT. *RASAL2*, which showed a highly significant gene-level association in MAGMA, encodes a RAS GTPase-activating protein that negatively couples to the ERK/MAPK cascade.^15^ Given that key periodontal pathogens – such as *Porphyromonas gingivalis* outer membrane vesicles – activate ERK1/2, JNK, and p38 MAPK in gingival epithelial cells and upregulate proinflammatory mediators including IL-6 and IL-843, we hypothesise that *RASAL2* variation modulates Ras–ERK/MAPK signalling to influence inflammatory amplification and tissue remodelling in periodontitis, nominating it as a priority effector gene. The second axis involves *C5orf27* or *SMIM3*, with *C5orf27* emerging as a top signal in MAGMA. Functional data indicate that *SMIM3* participates in PI3K–AKT signalling. SMIM3 knockdown decreases phosphorylated PI3K and AKT26. We infer that variation at *C5orf27* or *SMIM3* may influence immune tone and tissue homeostasis via the PI3K–AKT axis in the PME. This is consistent with observations that exosomes from periodontal ligament stem cells promote macrophage M1 polarisation by inhibiting PI3K/AKT and activating NF-κB–an effect reversed by PI3K activation.^46^ In addition, a high-confidence long non-coding RNA identified by TWAS, *RP11-563N4.1*, points to an additional regulatory pathway. Multiple studies show that lncRNAs influence bone loss in periodontitis by regulating immune cell composition, proinflammatory cytokine secretion, and bone metabolism. For example, loss of lncR-APDC aggravates bone loss, which can be reversed by local supplementation.^53^ However, *RP11-563N4.1* has not been functionally characterised in periodontal tissue, and no functional similarity to lncR-APDC has been established. Its identification as a high-confidence effector by both TWAS and FOCUS nonetheless marks it as a priority candidate. Whether it participates in immune–bone metabolism crosstalk remains an open question that warrants dedicated functional characterisation in future work. In summary, our priority validation list includes *RASAL2*, *C5orf27/SMIM3*, and *RP11-563N4.1*. These candidates would benefit from future functional studies – for example, combined gene-perturbation and pathway-modulation experiments in periodontal-relevant cell types and *in vivo* models – to assess their effects on cytokine profiles, osteoclast activity, and tissue remodelling.

Our single-cell enrichment analysis revealed consistent genetic enrichment across endothelial cells, pericytes, and smooth muscle lineages in multiple organs. This cross-tissue vascular–smooth muscle–matrix signal aligns with independent observations of endothelial inflammatory activation in the gingival microenvironment, suggesting that genetic susceptibility to periodontitis may, in part, reflect a systemic sensitivity at the microvascular–immune interface.^40^ Multiple independent lines of evidence support a vascular–matrix axis. First, enrichment in vessel-wall lineages mirrors the pathology of increased vascular permeability and enhanced transendothelial leukocyte migration in periodontitis. Mechanistically, endothelial NAMPT–FASN–driven lipogenesis can downregulate VE-cadherin and disrupt barrier function.^48^ Second, a neurovascular–immune axis provides upstream control of endothelial permeability and immune extravasation. The sensory neuropeptide substance P promotes vasodilation and permeability through endothelial *NK1/Tacr1* receptors, thereby facilitating inflammatory cell infiltration.^21^ Third, enrichment in pericyte-related lineages may reflect differential susceptibility in repair and bone remodelling, as CD146+/NG2+ pericytes migrate to the alveolar bone surface and contribute to osteogenesis during both disease progression and healing. Their migration and osteogenic activity are driven by the CXCL12–CXCR4–Rac1 pathway and exert functional effects on bone mass.^2^ Collectively, our cross-organ enrichment across endothelial, pericyte, smooth muscle, and matrix lineages, supported by independent evidence, delineates a testable vascular–matrix axis whose core pathological nodes include endothelial inflammatory activation and barrier permeability, neurovascular–immune upstream regulation, and pericyte-mediated repair and bone remodelling. This framework resonates with the central role of VEGF-mediated angiogenesis and permeability in periodontitis and highlights candidate directions for future investigation, such as endothelial barrier integrity, NAMPT–FASN-mediated lipogenesis, NK1/Tacr1 signalling, and the CXCL12–CXCR4–Rac1 axis, whose relevance to periodontal pathobiology will require dedicated functional study.^31,37
^


Finally, spatial localisation within a fetal tissue atlas revealed enrichment of genetic signals in the lung. This finding is consistent with the biology of the oral–lung mucosal axis, wherein the oropharyngeal microbiome is a major determinant of the pulmonary microbiome.^28^ In the context of our PME traits anchored by *Porphyromonas*, existing evidence shows that *Porphyromonas gingivalis* can disseminate to and colonise lung tissue, become enriched in bronchoalveolar lavage fluid, and reshape the pulmonary microbiome, thereby aggravating chronic obstructive pulmonary disease.^10^ Some studies in bronchiectasis further demonstrate overrepresentation and co-residence of anaerobic genera closely linked to periodontal ecology – such as *Prevotella*, *Fusobacterium*, and *Porphyromonas* – accompanied by differences in clinical phenotypes. These observations support the interpretation that Bacteroidales-related genetic signals may exert their effects by influencing airway colonisation and host responses.^30^ From a translational perspective, meta-analyses of randomised controlled trials indicate that oral hygiene interventions including chlorhexidine significantly reduce ventilator-associated pneumonia among mechanically ventilated patients.^52^ Accordingly, our findings suggest that optimising the oral microbiome and strengthening oral care may improve pulmonary outcomes along the oral–lung axis, providing genetic support for this clinical practice.

Several limitations should be noted. First, the six input GWAS varied substantially in sample size. The limited power of the microbial GWAS may reduce the precision of common-factor estimation and attenuate their contribution to the latent factor. Second, all input GWAS were restricted to individuals of European ancestry, so the generalisability of our findings to other populations remains to be established. Third, the cell-type and spatial enrichment analyses relied on mouse single-cell and spatial transcriptomic atlases mapped to human genes through homolog conversion, and such cross-species extrapolation may not fully capture human-specific cell states or tissue contexts. Finally, the PRS was not validated in an independent cohort, and its predictive utility for periodontitis risk stratification will require evaluation in future external data sets.

In conclusion, by jointly modelling six periodontal and oral microbial phenotypes, this study delineates a shared genetic architecture centred on vascular–matrix biology that complements and extends current periodontal genetics. Our findings are concordant with recent evidence implicating impaired tissue maintenance and repair as causal mechanisms in periodontitis, particularly along the angiogenesis and extracellular matrix axes, while adding a cross-phenotype, systems-level perspective that single-trait analyses cannot provide. Clinically, the prioritised pleiotropic loci and candidate effector genes offer genetically informed substrates for risk stratification and for the development of mechanism-guided preventive and therapeutic strategies. Realising this translational potential will require validation in diverse ancestral populations and functional confirmation of the prioritised candidates.

### Acknowledgements

We gratefully acknowledge the original authors for providing the data sets used in this research.

#### Ethics approval and consent to participate

This study used GWAS data from prior research. Ethical approvals and consents were obtained in the original studies.

#### Availability of data and materials

All data sets analysed in this study are publicly available. Summary statistics for gingivitis (GCST90478256), periapical abscess (GCST90478248), order Bacteroidales (GCST90429802), and genus Porphyromonas (GCST90429816) were obtained from the NHGRI-EBI GWAS Catalog (https://www.ebi.ac.uk/gwas/). Summary statistics for chronic periodontitis and dental calculus were obtained from the FinnGen Release 11 (https://www.finngen.fi/en). Accession details for each trait are also provided in Table S1.

#### Competing interests

The authors declare that they have no competing interests.

#### Funding

This study was supported by the Hunan Provincial Natural Science Foundation of China (Grant No. 2025JJ83419).

#### Supplementary material

To view the supplementary material for this manuscript, please contact the authors directly at 18874731873@163.com.

## References
